# Impact of Mental Health Screening on Promoting Immediate Online Help-Seeking: Randomized Trial Comparing Normative Versus Humor-Driven Feedback

**DOI:** 10.2196/mental.9480

**Published:** 2018-04-05

**Authors:** Isabella Choi, David N Milne, Mark Deady, Rafael A Calvo, Samuel B Harvey, Nick Glozier

**Affiliations:** ^1^ Brain and Mind Centre Central Clinical School, Sydney Medical School University of Sydney Sydney Australia; ^2^ Faculty of Engineering and Information Technologies University of Technology Sydney Sydney Australia; ^3^ Wellbeing Technology Lab School of Electrical and Information Engineering The University of Sydney Sydney Australia; ^4^ Black Dog Institute University of New South Wales Sydney Australia

**Keywords:** online help-seeking, screening, feedback, randomized trial, mental health, resilience, depression

## Abstract

**Background:**

Given the widespread availability of mental health screening apps, providing personalized feedback may encourage people at high risk to seek help to manage their symptoms. While apps typically provide personal score feedback only, feedback types that are user-friendly and increase personal relevance may encourage further help-seeking.

**Objective:**

The aim of this study was to compare the effects of providing normative and humor-driven feedback on immediate online help-seeking, defined as clicking on a link to an external resource, and to explore demographic predictors that encourage help-seeking.

**Methods:**

An online sample of 549 adults were recruited using social media advertisements. Participants downloaded a smartphone app known as “Mindgauge” which allowed them to screen their mental wellbeing by completing standardized measures on Symptoms (Kessler 6-item Scale), Wellbeing (World Health Organization [Five] Wellbeing Index), and Resilience (Brief Resilience Scale). Participants were randomized to receive normative feedback that compared their scores to a reference group or humor-driven feedback that presented their scores in a relaxed manner. Those who scored in the moderate or poor ranges in any measure were encouraged to seek help by clicking on a link to an external online resource.

**Results:**

A total of 318 participants scored poorly on one or more measures and were provided with an external link after being randomized to receive normative or humor-driven feedback. There was no significant difference of feedback type on clicking on the external link across all measures. A larger proportion of participants from the Wellbeing measure (170/274, 62.0%) clicked on the links than the Resilience (47/179, 26.3%) or Symptoms (26/75, 34.7%) measures (χ^2^=60.35, *P*<.001). There were no significant demographic factors associated with help-seeking for the Resilience or Wellbeing measures. Participants with a previous episode of poor mental health were less likely than those without such history to click on the external link in the Symptoms measure (*P*=.003, odds ratio [OR] 0.83, 95% CI 0.02-0.44), and younger adults were less likely to click on the link compared to older adults across all measures (*P*=.005, OR 0.44, 95% CI 0.25-0.78).

**Conclusions:**

This pilot study found that there was no difference between normative and humor-driven feedback on promoting immediate clicks to an external resource, suggesting no impact on online help-seeking. Limitations included: lack of personal score control group, limited measures of predictors and potential confounders, and the fact that other forms of professional help-seeking were not assessed. Further investigation into other predictors and factors that impact on help-seeking is needed.

**Trial Registration:**

Australian New Zealand Clinical Trials Registry ACTRN12616000707460; https://www.anzctr.org.au/ Trial/Registration/TrialReview.aspx?id=370187 (Archived by WebCite at http://www.webcitation.org/6y8m8sVxr)

## Introduction

Mental health screening and feedback has been purported to improve recognition and encourage service use, despite minimal evidence supporting its benefits in the community [1]. Mental health screening websites and mobile apps are widely available, and many provide personal feedback related to mood, anxiety, and wellbeing [2,3]. While personal feedback is often incorporated in online and mobile interventions as an engagement strategy [4], few studies have examined whether providing such feedback encourages help-seeking in brief online screening tools. Online mental health screeners with personal feedback appear to engage participants, with a third of participants completing one or more follow-ups after initial screening and feedback [5]. There is some support from observational studies that providing personal feedback encourages help-seeking; for instance, 42% of university students who received positive screening results after using a self-help mental health screening website requested a referral to the university’s mental health clinic [6]. Similarly, BinDimh et al [7] provided personal score feedback in a depression-screening app and recommended that users with scores above threshold seek help from a health care professional. Approximately 38% of users who did not have a previous self-reported depression diagnosis reported that they had consulted a health care professional after one month [7]. However, only one randomized controlled trial has been conducted to evaluate whether providing personal score feedback after online screening promotes help-seeking from professional sources [8]. A large online sample was randomized to receive feedback about their mental health and information about treatment services, or receive no feedback, after completing a lengthy mental health survey. Participants who received feedback were significantly less likely to complete the follow-up measures about help-seeking after three months [8]. Among those who responded, there was no effect of depression feedback, and social anxiety feedback appeared to have a small negative effect on help-seeking. However, the differential completion rates among those who received feedback or not indicate issues with attrition and potential nonresponse bias. Overall there is mixed evidence to support the effects of online screening, but all studies to date have only focused on seeking professional help. Given the provision of online screening and feedback, it remains unclear whether there is an impact on online help-seeking.

Furthermore, the reason for differences in rates of help-seeking may be related to how the personal feedback is presented. Providing user-friendly and easily comprehensible information may be more useful than simply providing score feedback, as it increases the personal relevance of messages and subsequently increases the likelihood of deeper processing and strengthens motivation for behavior change [9]. There are many variations in which personalized feedback can be presented to enhance processing. Normative comparison of an individual’s results to a reference group is one of the widely used strategies to increase salience of the message. Normative feedback is effective in reducing problematic drinking behaviors as it reveals discrepancies in individual behavior, along with perceived and actual group behavior [10]. Providing normative feedback for mental health may also improve help-seeking among those with high scores. Indeed, a qualitative study reported that the majority of undergraduate students with moderately severe to severe depressive symptoms found that receiving normative feedback increased their awareness about their own symptoms and motivated them to seek treatment [11]. Another potential way of engaging respondents is through the use of humor in the feedback messages. Self-stigma of mental illness is associated with low self-esteem [12] and deters help-seeking [13,14]. However, it has been found that people with mental illness who view their illness in a relaxed and humorous way have higher self-esteem [15]. Indeed, humor has been used as a successful strategy to engage Australian men in mental health issues [16] and to reduce mental health stigma among military personnel [17], and may be a useful feedback tool to reduce stigma and encourage help-seeking.

The current study describes the results of a pilot randomized trial that compared the impact of receiving personal normative versus humor-driven feedback on promoting immediate online help-seeking. This study attempted to address the gaps in existing literature by assessing online help-seeking rather than face-to-face help, and to examine immediate help-seeking to avoid loss to follow-up.

## Methods

### Participants and Procedure

Participants were recruited on social media websites between June-October 2016. A series of paid advertisements were placed on Facebook mobile with themes such as, “worried about your mental health?” “how tough is your mind,” and, “are you on the path to happiness?” Partner organizations (beyondblue, the Black Dog Institute, and the Movember Foundation) also shared posts about the study on their Facebook and Twitter pages. Interested individuals were directed to the study website or the Google Play or Apple App Store to download the *Mindgauge* app for free, which featured measures on *Symptoms*, *Resilience*, and *Wellbeing*. Individuals were eligible to participate if they were 18 years or older, owned a smartphone, and were a resident in Australia, New Zealand, the United States, or the United Kingdom. Informed consent to take part in this study was obtained when participants used the app for the first time.

Participants first completed basic demographic questions on gender, age, and whether or not they had a self-reported period of poor mental health for more than one month in the past two years. Participants were then free to choose to complete any of the measures on *Symptoms*, *Resilience*, and *Wellbeing*. Users could complete the measures more than once (following a one-week gap), but for the purposes of this study only the first completion of each measure was analyzed because seeking help after subsequent completions of the measure may indicate heightened interest or concern in that measure rather than the impact of the feedback.

### Randomization

**Figure 1:**
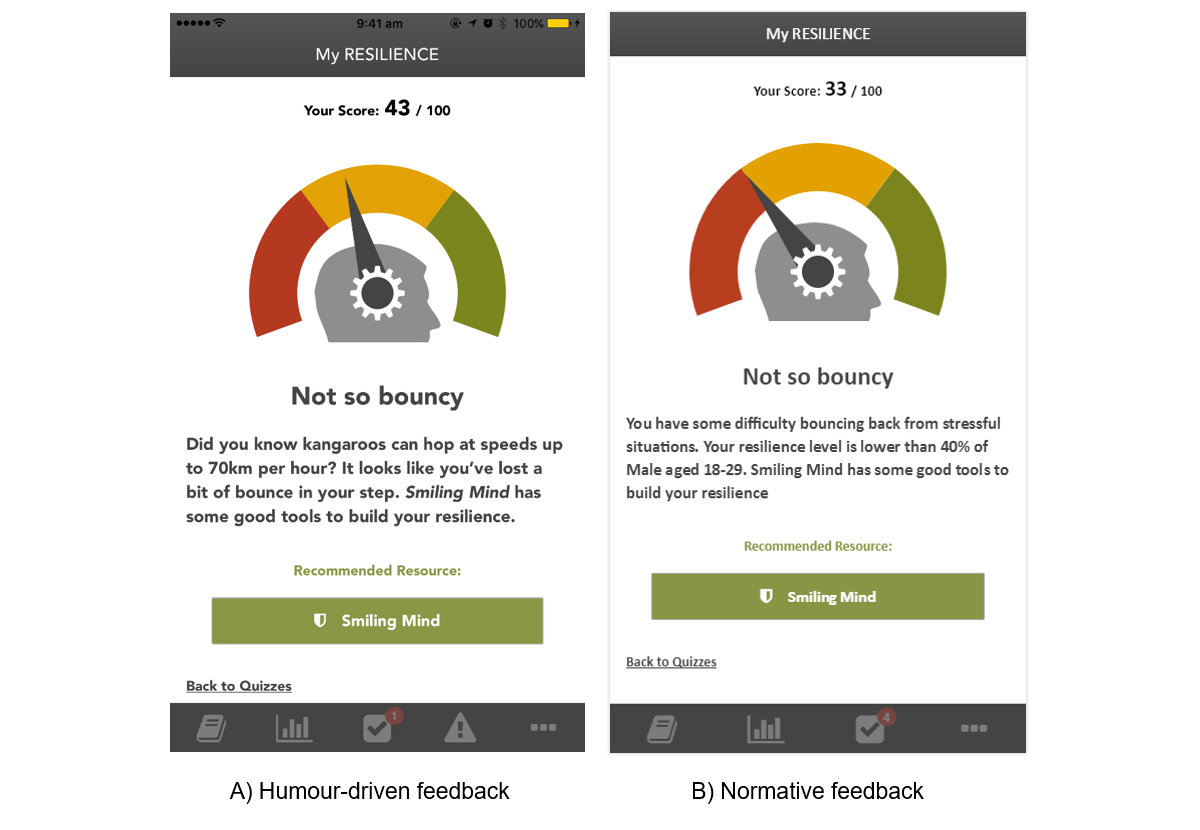
Screenshot of the feedback for moderate resilience.

Upon completion of each measure, participants were randomly allocated to receive either (1) normative feedback comparing their scores to a relevant reference group, or (2) humor-driven feedback that presented their scores in a light-hearted manner (see Figure 1 for an example). Randomization was independent for each measure (ie, a participant was randomized for the *Symptoms* measure and randomized again for the *Resilience* measure). The humor-driven feedback was pilot tested among the larger research team. The feedback messages were slightly different depending on the score range and the list of feedback for each measure is shown in Multimedia Appendix 1.

**Figure 2:**
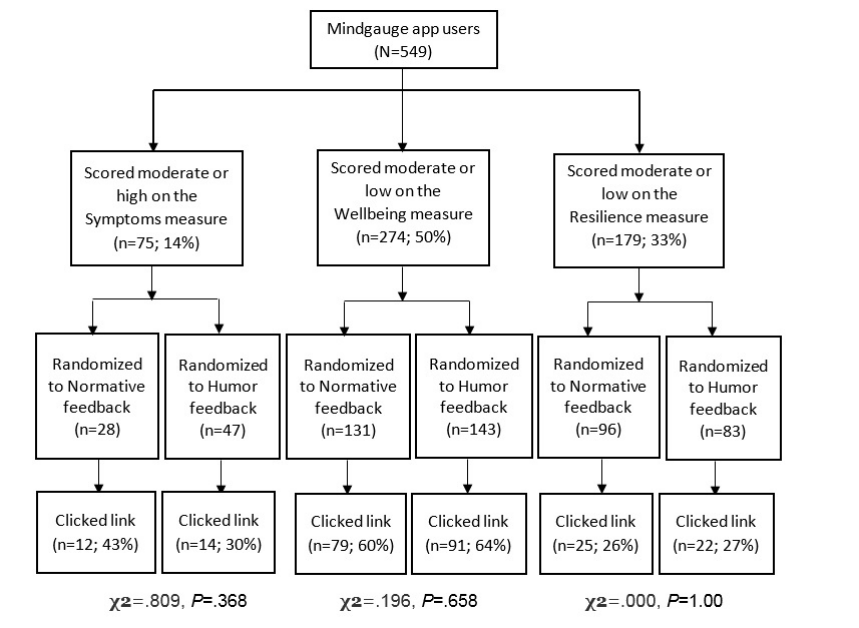
Participant flow in trial.

Participants received feedback immediately after completing each measure. To assess the impact of the type of feedback on immediate online help-seeking, participants who scored within the moderate or poorer categories of any measure (as described below) were additionally provided with a link to an appropriate external online resource and were included in the analyses. Figure 2 shows the flow of participants.

### Measures

#### Primary Outcome

The study website automatically recorded whether participants clicked on the link to the online resource presented as part of their feedback, as a proxy of online help-seeking.

#### Self-Reported Measures

##### Symptoms

The Kessler 6-item Scale [18] is a measure of nonspecific psychological distress and is validated for use among the Australian population. Participants with scores ranging from 12-19 were considered to have moderate symptoms, and scores from 20-30 were high symptoms, based on standardized cut-points [19].

##### Wellbeing

The World Health Organization (Five) Well-Being Index [20] is a commonly used measure of subjective wellbeing. Five items produce a score ranging from 0 to 25, with higher scores indicating better quality of life. Using the population mean as the center, scores between 0-12 were considered as low wellbeing, and scores between 13-21 were considered moderate wellbeing.

##### Resilience

The Brief Resilience Scale [21] measures one’s ability to bounce back from difficult times. Scores range from 6 to 30, with higher scores indicating better resilience. Similarly, using the population mean as the center, scores between 6-17 were considered as low resilience while scores between 18-24 were considered moderate resilience.

### Statistical Analysis

Results were analyzed using IBM SPSS 24 statistical software. Chi-square tests were used to compare the proportion of participants who clicked on a link between the normative and humor-driven feedback conditions for each measure, and to compare the difference in clicks among the measures. Logistic regression analyses were used to examine the association between clicks on the link and demographic factors for each measure independently and pooled.

### Ethical Approval

The study was approved by the Human Research Ethics Committee at the University of New South Wales (HC15584).

## Results

### Participant Characteristics

Of the 549 unique *Mindgauge* app users, 318 participants scored in the moderate or poorer ranges on one or more measures and were included in the analyses, with 161 participants (161/549, 29.3%) having scored undesirably on one measure, 104 (104/549, 18.9%) on two measures, and 53 (53/549, 9.7%) on all three measures. Over half of the included sample (197/318; 61.9%) was female, 118 (118/318, 37.1%) were male, and 3 (3/318, 0.9%) did not specify their gender. There were 93 participants (93/318, 29.2%) aged between 18-29 years, 79 (79/318, 24.8%) aged between 30-39 years, 98 (98/318, 30.8%) aged between 40-49 years, and 48 (48/318, 15.1%) aged 50 or above. More than two thirds of participants (228/318, 71.7%) reported that they had an episode of poor mental health in the past.

### Clicks on Links

There was no significant impact of feedback type on whether participants clicked on the external link for each of the measures (all *P* values >.05; Figure 1). A significantly higher proportion of participants who scored below threshold on the *Wellbeing* measure (170/274, 62.0%) clicked on the links compared to those who scored undesirably on the *Resilience* (47/179, 26.3%) or *Symptoms* (26/75, 34.7%) measures (χ^2^=60.35, *P*<.001).

### Factors Associated With Clicking on the Link

Logistic regression analyses found that participants with previous poor mental health were less likely than those without such history to click on the link in the *Symptoms* measure (*B*=–2.48, Wald=8.54, *P*=.003, odds ratio [OR] 0.83, 95% CI 0.02-0.44). There were no significant demographic factors associated with clicking on the link for the *Wellbeing* or *Resilience* measures. When all three measures were pooled, participants aged 18-29 were significantly less likely to click on the link compared to those above 50 years of age (*B*=–.82, Wald=7.78, *P*=.005, OR 0.44, 95% CI 0.25-0.78).

## Discussion

This pilot randomized trial showed no significant difference between normative and humor-driven feedback on the likelihood of an individual who has screened positive for a poor mental health outcome clicking through to online resources to seek further help. There was no evidence to suggest that the manner in which personal feedback was presented encouraged individuals to seek treatment, suggesting that there may be other factors influencing whether one seeks help after receiving personal feedback, which warrant further investigation. These factors could be related to personal characteristics or other external factors, such as stages of change, motivation, or the perceived credibility of feedback or helpfulness of an intervention. Web-based and smartphone app interventions are often perceived as low in credibility and helpfulness, which are key considerations for patients in choosing to engage with mental health treatment [22]. Given that there is support suggesting that providing simple information about the intervention improves attitudes towards Internet interventions and intention to use [23,24], it is possible that the rate of clicks to resources in this study may be improved if we provide further information about those resources in the feedback.

Nonetheless, the online help-seeking rate in our study ranged from 26% to 60% and was comparable to the rates of seeking face-to-face help following online screening, as previously reported [6-8]. Interestingly, the *Wellbeing* measure had more frequent clicks than the *Symptoms* or *Resilience* measures regardless of feedback type. It is possible that online resources aimed at improving “wellbeing” were seen as more attractive or achievable than improving “symptoms” or “resilience,” which may have a negative connotation related to poor mental health. It is also possible that receiving negative personal feedback on the *Symptoms* and *Resilience* measures may have been confronting and inadvertently exacerbated avoidance behaviors [8].

The finding that individuals with previous poor mental health were less likely to click on external resources on the *Symptoms* measure suggested that they may be using screening apps for symptom monitoring rather than treatment seeking. Conversely, this finding provides some support that such screening tools may be targeted to those who were distressed, but without a history of mental health problems, to improve recognition and treatment seeking. However, this finding should be interpreted with caution given the small numbers in the *Symptoms* measure, and it was not significant when all measures were pooled. Younger people were also less likely to click on the link across all measures compared to the oldest age group. This result is in line with previous studies showing that there is a lack of evidence that online services facilitate mental health help-seeking in young people [25].

A strength of this study was that it measured clicks to an online resource as a proxy of immediate online help-seeking. The use of objective measures of help-seeking within the app overcame some of the limitations in previous studies, such as reliance on participant self-report and loss to follow-up [4,5], which may have led to nonresponse sampling bias. However, it is important to note that clicks to the online resource only suggest interest in seeking further help online, but do not indicate actual engagement in further online help-seeking. Participants may have also engaged in other forms of help-seeking using other online resources and treatments, or sought face-to-face help, but this was not assessed. Future studies could also explore reasons why participants did not seek further help and explore longer-term outcomes. Another limitation of the study was the lack of a personal score control group; as such, we were unable to determine if there were any added effects of normative and humor-driven feedback to simply providing personal scores. Furthermore, there were limited measures of predictors and we were unable to control for potential confounders, such as self-esteem and stigma. A limitation in the app build and design meant that scores were calculated within the app but were not recorded on the database; thus, we were unable to report the psychometric properties of the measures and conduct further analyses to explore whether severity of scores impacted on clicking on the link. Despite pilot testing the humor-driven feedback, humor perception is subjective and not necessarily transcultural, and thus may be misunderstood or even be seen as trivializing the matter of mental health. However, there is support that the use of humor as a communication tool in medical contexts has a small but positive effect on perceived credibility [26], and our results suggest that the humor-driven feedback used in this study did not appear to negatively impact help-seeking.

This is the first study to compare the impact of different types of feedback on seeking online mental health support. The nil findings suggest that the feedback type does not affect online help-seeking, and less frequent clicks on the *Resilience* and *Symptoms* measures echo previous studies that indicate that feedback on certain measures may be less conducive to help-seeking [8]. Nonetheless, the 60% click rate on the *Wellbeing* measure provides encouraging support that online screening tools can promote help-seeking. Given the widespread use of online and mobile screening tools, and the limited research on its efficacy, further research is needed to explore predictors and factors that improve help-seeking, such that developers and researchers can better tailor such tools to address the gaps in service use.

## Appendix

Multimedia Appendix 1 Normative and humor-driven feedback provided for scores on the measures.

## Abbreviations

OR: odds ratio
