# Supplementary material for: Impact of Mental Health Screening on Promoting Immediate Online Help-Seeking: Randomized Trial Comparing Normative Versus Humor-Driven Feedback
Source: JMIR Ment Health. 2018 Apr 5;5(2):e26. doi: 10.2196/mental.9480 (PMC5938694; doi:10.2196/mental.9480)
Supplement: Multimedia Appendix 1 [file mental_v5i2e26_app1.pdf]

Multimedia Appendix 1. Normative and humor-driven feedback provided for scores on the measures.

Measure	Category (Score)	Normative condition:	Humour-driven condition
**Symptoms (K6)**	**Low**<br><br>**(6-11)**	Doing well:<br><br>Your mood was well over the past week. Your mood was higher than XX% of (insert gender) aged (insert age group). Keep it up!	Doing well:<br><br>Looks like you're kicking life's arse right now! Your mood scores show that you've been feeling good over the past week. Keep it up!
	**Moderate**<br><br>**(12-19)**	Watch out:<br><br>You've been feeling a bit low and anxious over the past week. Your mood was lower than XX% of (insert gender) aged (insert age group). You could get instant help from our online program My Compass to improve the way you feel.	Watch out:<br><br>Sometimes we hit a bit of a rough patch, hey, even the Mona Lisa needs upkeep now and then. If you're looking for an upgrade, try our online program MyCompass for instant online help.
	**High**<br><br>**(20-30)**	Time to take action:<br><br>You've been feeling depressed and anxious over the past week. Your mood was lower than XX% of (insert gender) aged (insert age group). There are a range of treatments that can help these types of symptoms. You could speak to your GP or get instant help from our online program My Compass"	Time to take action:<br><br>Life can be a real rollercoaster sometimes and it seems like you've hit a bump. As a humble tracking app, there's not a whole lot I can do to upgrade your system. However, I do have some very capable friends. Try My Compass - it can point you to real solutions. Or if you prefer someone in real life, speak to your GP.

**Wellbeing (WHO-5)**	**Low**<br><br>**(0-12)**	Room for improvement:<br><br>Your wellbeing score shows that you haven't been feeling healthy and happy lately. Your wellbeing was lower than XX% (insert gender) aged (insert age group). There are lots of practical things we can do to improve our wellbeing - learn more at Daily Challenge.	Room for improvement:<br><br>What do humans and dung beetles have in common? We both feel crap sometimes. Life can wear us down, but there are lots of practical things we can do to improve our wellbeing - learn more at Daily Challenge (no advice for dung beetles)
	**Moderate (13-21)**	Feeling ok:<br><br>Your wellbeing levels are fair. Your score was lower than XX% of (insert gender) aged (insert age group). Check out the Daily challenge for practical tips to improve your wellbeing.	Feeling ok:<br><br>Sounds like your daily life can sometimes be a daily grind, check out the Daily Challenge for a whole load practical ways to improve things…daily.
	**High**<br><br>**(22-25)**	Feeling great:<br><br>Your wellbeing levels are great. Your score was higher than XX% of (insert gender) aged (insert age group). Well done!	Feeling great:<br><br>Feeling healthy and happy about yourself is like being outside when the weather is just right. Looks like the weather has been perfect for you!
**Resilience (BRS)**	**Low**<br><br>**(6-17)**	Feeling flat:<br><br>It seems you get overwhelmed by stressful events. Your resilience is lower than XX% of (insert gender) aged (insert age group). Smiling Mind has some good tools to build your resilience.	Feeling flat:<br><br>It’s not unusual to feel like a basketball that’s gone flat, get your bounce back at Smiling Mind. It has some good tools to build your resilience.

**Moderate (18-24)**	Not so bouncy:<br><br>You have some difficulty bouncing back from stressful situations. Your resilience level is lower than XX% of (insert gender) aged (insert age group). Smiling Mind has some good tools to build your resilience.	Not so bouncy:<br><br>Did you know kangaroos can hop at speeds up to 70km per hour? It looks like you've lost a bit of bounce in your step. Smiling Mind has some good tools to build your resilience.
**High**<br><br>**(25-30)**	Bouncing back well:<br><br>You are well equipped to bounce back from stress. You are more resilient than XX% of (insert gender) aged (insert age group).	Bouncing back well:<br><br>Looks like you'd bounce back from a black hole! Well done coping with stress, keep it up!
